# Uncertainty about old information results in differential predator memory in tadpoles

**DOI:** 10.1098/rspb.2023.0746

**Published:** 2023-05-10

**Authors:** Adam L. Crane, Gabrielle H. Achtymichuk, Ita A. E. Rivera-Hernández, Alexyz A. Preagola, Himal Thapa, Maud C. O. Ferrari

**Affiliations:** ^1^ Department of Biology, University of Saskatchewan, Saskatoon, SK, Canada; ^2^ Department of Veterinary Biomedical Sciences, University of Saskatchewan, Saskatoon, SK, Canada

**Keywords:** alarm cues, conditioning, learning, predator recognition, retention

## Abstract

As information ages, it may become less accurate, resulting in increased uncertainty for decision makers. For example, chemical alarm cues (AC) are a source of public information about a nearby predator attack, and these cues can become spatially inaccurate through time. These cues can also degrade quickly under natural conditions, and cue receivers are sensitive to such degradation. Although numerous studies have documented predator-recognition learning from fresh AC, no studies have explored learning from aged AC and whether the uncertainty associated with this older information contributes to shortening the retention of learned responses (i.e. the ‘memory window’). Here, we found that wood frog tadpoles, *Lithobates sylvaticus*, learned to recognize a novel odour as a predator when paired with AC aged under natural conditions for up to 1 h. However, only tadpoles conditioned with fresh AC were found to retain this learned response when tested 9 days after conditioning. These results support the hypothesis that the memory window is shortened by the uncertainty associated with older information, preventing the long-term costs of a learned association that was based on potentially outdated information.

## 1. Introduction

To maximize fitness, animals must make correct decisions, such as in balancing their foraging needs with predator avoidance [1]. A prerequisite for making an optimal decision, however, is for an individual to first correctly detect and recognize pieces of information (i.e. cues) in their environment. Hence, animals have evolved highly specialized sensory systems for the acquisition of various information types (e.g. visual, auditory and chemical) [2]. However, complete knowledge of the environment is generally not attainable as there are often informational limitations (e.g. incompleteness, unreliability or context dependency) which consequently hinder the expression of appropriate behavioural responses [3]. For instance, a predator cue may indicate danger, but that information may only be valid at a particular time or in a particular location. The prey would thus need additional information about the timing and directionality of the predator cue [4]. Such informational limitations result in uncertainty, which can lead to decision errors [5]. Such errors may include displaying a weaker response or switching to a different type of response when uncertain about whether a full reaction is optimal or unnecessary costly in terms of energetic losses and missed opportunities [6].

Chemosensory cues provide a rich source of public information about the environment [7]. Biotic sources of chemical cues include heterospecifics (e.g. kairomones of predators or prey) as well as conspecifics (e.g. members of a social group). Cues from conspecifics, in particular, have a wide range of functions, serving as crucial sources of information in antipredator decisions (e.g. in insects [8]), as well as in decisions related to foraging (e.g. in mammals [9]), reproduction (e.g. in reptiles [10]) and territorial defence (e.g. in crustaceans [11]). There are two basic categories of social chemicals used in risk assessment; ‘alarm cues’ (AC) are released by injured conspecifics (generally during a predator attack), whereas ‘disturbance cues’ are characterized as chemical cues released by individuals that are disturbed but uninjured [12,13]. Both cues appear to be particularly widespread among aquatic species, perhaps due to the solvent properties of water or the limitation of visual information in some aquatic environments (e.g. due to structural barriers or turbidity). However, between the two types of cues, AC function as the more reliable source of information about predation risk because they are released only upon tissue damage (e.g. skin and muscle [14]) whereas disturbance cues can be released upon any type of disturbance (biotic or abiotic). Perhaps the higher reliability of AC explains the widespread and rapid nature of AC learning of predator recognition among aquatic taxa (e.g. in flatworms [15], mosquitos [16], crayfish [17], fish [18] and amphibians [19]). Such learning typically requires only a single experience in which AC (representing predation risk) are paired with other cues in the area such as the sight or odour of a novel species (representing the cause of the released AC) [20].

Chemical cues that are initially reliable can become inaccurate over time. Old AC, for instance, can convey inaccurate information about the location of a predator if they have been moved by currents, or the predator has left the area. This has led researchers to explore the active time of AC (i.e. the rate of attenuation) [21]. Studies show that, indeed, AC degrade and lose their effectiveness over time (i.e. they no longer elicit antipredator responses in cue receivers). In one study, AC from amphipods (*Gammarus lacustris*) and fish (*Pimephales promelas*) were found to evoke weaker antipredator responses after 3 h of ageing compared to no responses after 6 h of ageing [22]. That study aged the AC under laboratory conditions at room temperature, whereas other studies have documented more rapid attenuation, particularly under natural conditions (e.g. solar radiation, temperature fluctuations and microbiota) [23]. For example, under natural conditions, AC from wood frog tadpoles, *Lithobates sylvaticus*, were significantly less effective in eliciting antipredator responses when tested after 2 h of ageing, whereas they were completely ineffective after 4 h of ageing [24]. These studies reveal that as AC age (i.e. undergo chemical degradation), the content of the information also changes. The prey stop responding to the cues, either because the cues are no longer detected, or alternatively because they are still detected but are perceived as old and no longer reliable.

Previously, we presented a theoretical framework predicting that the uncertainty associated with a learned cue should affect the length of time that responses to that cue are retained (i.e. the length of the ‘memory window’) [25]. Specifically, as uncertainty about the informative value of a cue increases, individuals should devalue the cue and eventually ignore it completely. Hence, their memory window decreases. Evidence for such a pattern comes from AC learning studies on wood frog tadpoles [25,26]. In one study, tadpoles were conditioned to recognize a predator odour (PO) either 1, 2 or 4 times; more conditionings equated to less uncertainty about the predator's identity. On the following day, tadpoles exposed to the PO displayed learned antipredator responses that were similar in intensity regardless of the number of conditionings. However, for tadpoles tested 11 days after conditioning, only the individuals that had received multiple conditionings (i.e. less uncertainty) continued to display the response (i.e. their memory window was longer). These findings suggest that, at least in some cases, the memory window may serve as a proxy for measuring uncertainty [26]. While this previous work manipulated uncertainty that was associated with AC learning by changing the total amount of information available to prey, no studies to date have explored the age (i.e. degradation) of AC as a source of uncertainty affecting the memory window.

In this study, we explored how uncertainty associated with old information might affect the memory window in wood frog tadpoles. Upon hatching, these tadpoles face intense predation pressure (e.g. Odonata, salamanders and birds) [27], and only a small percentage of individuals survive to adulthood [28]. Hence, there is strong selection for fine-tuned antipredator strategies [1]. These tadpoles reduce activity in response to predation risk [29] and have demonstrated sophisticated learning processes (e.g. [30,31]). Here, we conducted two experiments during which tadpoles were given an opportunity to learn predator recognition from AC that were aged under natural conditions for different lengths of time (fresh, 30 min or 60 min). In each case, the AC were paired with a novel PO during a conditioning phase, whereas a control group received a sham conditioning with blank water. In experiment 1, we tested the initial learned responses of tadpoles 2-day post-conditioning, exposing them to either the conditioned odour or water (control) and measuring their antipredator response (electronic supplementary material, figure S1). In experiment 2, we tested for the retention of learned responses 9-day post-conditioning (electronic supplementary material, figure S1). Based on the aforementioned studies, we hypothesized that AC would degrade quickly over time, resulting in uncertainty. Thus, we predicted that only tadpoles conditioned with fresh AC would display significant retention of learned responses. By contrast, we predicted that 30 min of ageing would result in an initial learned response that would quickly wane, whereas tadpoles would fail to learn from AC aged for 60 min.

## 2. Methods

### (a) Study species

We collected 18 clutches of wood frog eggs from ephemeral ponds in central Saskatchewan in May 2018. We then transported the eggs to the R. J. F. Smith Centre for Aquatic Ecology where we split each egg mass into two subclutches and raised them in separate outdoor pools (50 l). Each pool was filled with the facility's filtered dechlorinated water (hereafter ‘water’) and received an approximately 10% water change every other day throughout the study period. Tadpoles hatched approximately 1 week after collection and were fed a mix of naturally growing algae, commercial algae and alfalfa pellets. As tadpoles grew, the groups were split into additional outdoor pools. This laboratory colony provided test subjects for several experiments conducted throughout summer 2018, including this study.

### (b) Cues

To obtain a novel PO, we used three adult tiger salamanders, *Ambystoma mavortium*, from our laboratory stock colony. Each individual was moved into a container filled with 1 l of unfiltered facility water for 24 h. Then, the water samples were mixed and frozen in plastic bags until being thawed before use in conditioning.

We also prepared tadpole AC according to standard procedures approved by our university's Animal Research Ethics Board. Donors were placed in a mortar and euthanized with a blow to the head and rapid pulverization using a pestle (e.g. [32,33]). As in previous studies, we used physical euthanasia because chemical methods may interfere with the chemical nature of the stimuli [34]. To simulate natural conditions where AC degrade, we prepared ‘stimulus buckets’ (5 l capacity; 20 cm diameter; 18 cm height) filled with 1 l of pond sediment and 3 l of water. The sediment was obtained from a pond lacking wood frog tadpoles and was drained but not dried prior to use. Because we were concerned about pseudoreplication of a single stimulus bucket, we replicated the bucket a total of six times. The buckets were then positioned in the sun for approximately 4 h prior to the addition of AC for use in the conditioning treatments.

### (c) Conditioning treatment

We conditioned 105 tadpoles from each of six clutches (630 tadpoles total) when tadpoles were at stages 25–28 of their development (limb buds forming) [35]. First, we moved each group of 105 individuals into a shared pail and then pseudo-randomly moved tadpoles in groups of five into plastic cups (21 cups with five tadpoles each = 105 individuals) filled with approximately 400 ml of water. The cups were then placed inside plastic pails (four cups per pail), and 2 l of water was added to the pail (around the cups) to buffer ambient temperature fluctuations. After five pails were full (i.e. 20 cups), the remaining cup was placed inside a sixth pail that was intended to counterbalance the potential effect of conditioning time (described below). The three surrounding cups in the sixth pail contained water but no tadpoles (electronic supplementary material, figure S2a). Thus, each clutch provided tadpoles for six pails (36 pails total), each of which would receive conditioning cues from one of the six stimulus buckets (electronic supplementary material, figure S2a). Within each pail, we assigned each cup to one of the four conditioning treatments where tadpoles would be exposed the following day to the novel PO paired with either fresh AC, 30 min old AC, 60 min old AC or blank water (a sham conditioning) from the stimulus bucket (electronic supplementary material, figure S2b). Each day during the study period, the tadpoles housed in the cups were given a 50% water change in the late afternoon before being fed with a piece of an alfalfa pellet.

After 1 day of acclimation in the conditioning cups, the conditioning treatments occurred between 14.25 and 15.55 (23 May 2018). First, we used a syringe to remove 120 ml of water from bucket 1, drawing water from the bottom of the bucket near the substrate and moving upwards through the water column as we filled two 60 ml syringes. Then, we used the water for sham conditionings, gently injecting 20 ml of the water paired with 5 ml of the novel PO into six cups (one from each clutch) assigned to bucket 1 (electronic supplementary material, figure S2a). After these sham conditionings, we added fresh AC (75 donors in 120 ml of water) to the stimulus bucket and let the cues disperse for 1 min. Then, we again removed 120 ml of water from bucket 1 and conditioned 1 cup from each clutch with 20 ml of fresh AC (used within 5 min of production) paired with 5 ml of the odour. This amount of AC (0.5 donors in 20 ml) in approximately 420 ml of water closely matches the concentrations used in previous studies on wood frog tadpoles exposed to conspecific AC (e.g. [36]). After 30 min, we again removed 120 ml of water from the stimulus bucket for the conditioning with 30 min old AC and repeated this process again after 60 min.

We staggered the timing of conditioning from stimulus buckets, where bucket 2 was used 5 min after bucket 1, and so on (electronic supplementary material, table S1). However, we were concerned about confounding variation in the time of conditioning (e.g. if the 60 min cues were always used later in the day than the fresh cues). To counterbalance any such effect, bucket 6 was used only for fresh AC conditionings at the end of the conditioning period (electronic supplementary material, figure S2a). However, we saw no effect of conditioning with fresh AC later in the day (*p* > 0.14 for all comparisons of bucket 1 versus bucket 6) and ignored the time of conditioning in our main analysis.

### (d) Experiment 1: initial learned responses at day 2

We planned to test for initial learned responses 1-day post-conditioning but instead conducted testing 2-day post-conditioning due to weather conditions. Testing occurred in plastic trays (68 × 40 cm area × 17.5 cm height) containing 15 test cups (0.5 l; 90% filled) that would hold one tadpole each. These tadpoles were gently poured into the test cups from the conditioning cups in a systematic order. Then, we added 2 l of water to each tray (around the testing cups) to buffer temperature changes before placement in the sun to promote swimming activity. Tadpoles then received 30 min of acclimation time before being tested by an observer who was blind to the treatments. Trials consisted of a 4 min pre-stimulus observation period, followed by a gentle injection of 5 ml of the testing stimulus (salamander odour or blank water), and a 4 min post-stimulus observation period. Each cup had a medial line that was used to quantify tadpole movement as the number of line crosses, recorded when the entire body of the tadpole had crossed the line. An assessment of reduced activity in response to the test cue requires that individuals be active during the pre-stimulus baseline period. Hence, in accordance with previous studies [34,37], tadpoles performing less than six line crosses during the pre-stimulus period were not tested further, resulting in unequal group sample sizes. In total, we tested 203 tadpoles (20–30 per treatment group).

### (e) Experiment 2: retention of learned responses at day 9

In experiment 2, we tested tadpoles that were not tested in experiment 1. These tadpoles remained in their conditioning cups for another week until being tested (9-day post-conditioning). All testing procedures matched those of experiment 1. However, fewer tadpoles were tested for antipredator responses because overall activity was much lower during testing on day 9 and a few were lost due to mortality or conditioning cup overflow (into the surrounding pails due to rainfall). Hence, sample sizes were lower for experiment 2 (149 tadpoles tested with 15–21 per treatment group).

### (f) Statistical analysis

For each experiment, we first confirmed that the pre-stimulus baseline data were similar across treatments (all *p* > 0.5) and then calculated the proportional change in line crosses [(post-pre)/pre]. Then for each experiment, we analysed the proportional change in line crosses using a general linear mixed model with the conditioning treatment (fresh AC, 30 min old AC, 60 min old AC or sham), the test cue (odour or water) and their interaction as fixed factors, and the clutch, the conditioning cup and the stimulus bucket as random factors. We used a Type I SS model due to the hierarchal structure of the experimental design where the conditioning cup was nested under the conditioning treatment and thus served as the unit of replication in the analysis [*Y* = treatment + cup (treatment) + clutch + bucket + cue + treatment × cue + error]. Because clutch and bucket were non-significant in both experiments (all *p* > 0.08), we decided to remove them from the final models [*Y* = treatment + cup (treatment) + cue + treatment × cue + error]. The family of the models was Gaussian, and we confirmed that the statistical assumptions (normally distributed residuals and homoscedasticity) were satisfied by inspection of residual plots. To interpret significant interactions, we split the data by the conditioning treatment and performed separate models for each group, testing the fixed effect of the test cue and the random effect of the conditioning cup [*Y* = cup + cue + error]. All analyses were performed in SPSS 23 with *α* = 0.05.

## 3. Results

### (a) Experiment 1: initial learned responses at day 2

**Figure 1.**
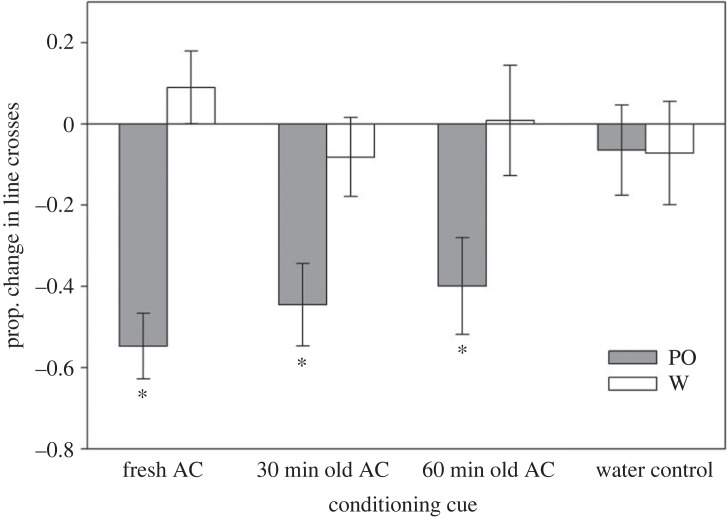
Mean (± s.e.) line crosses by tadpoles in experiment 1 (2-day post-conditioning) when tested with either the PO or blank water (W) after being conditioned with the odour paired with either fresh AC, 30 min old AC or 60 min old AC, or blank water. Asterisks represent significant test-cue comparisons, revealing reduced activity in response to the odour compared to testing with blank water.

The overall model revealed a significant interaction between treatment and cue where tadpoles in some conditioning treatments learned the odour as a threat (*F*_3, 79_ = 5.67, *p* = <0.001; figure 1; electronic supplementary material, table S2a). Tadpoles showed significant learned responses after being conditioned with fresh AC (*F*_1, 23_ = 38.65, *p* < 0.001; electronic supplementary material, table S2b), 30 min old AC (*F*_1, 18_ = 5.86, *p* = 0.026; electronic supplementary material, table S2c) and 60 min old AC (*F*_1, 21_ = 5.88, *p* = 0.024; electronic supplementary material, table S2d), but not with the water control (*F*_1, 17_ = 1.35, *p* = 0.26; electronic supplementary material, table S2e).

### (b) Experiment 2: retention of learned responses at day 9

**Figure 2.**
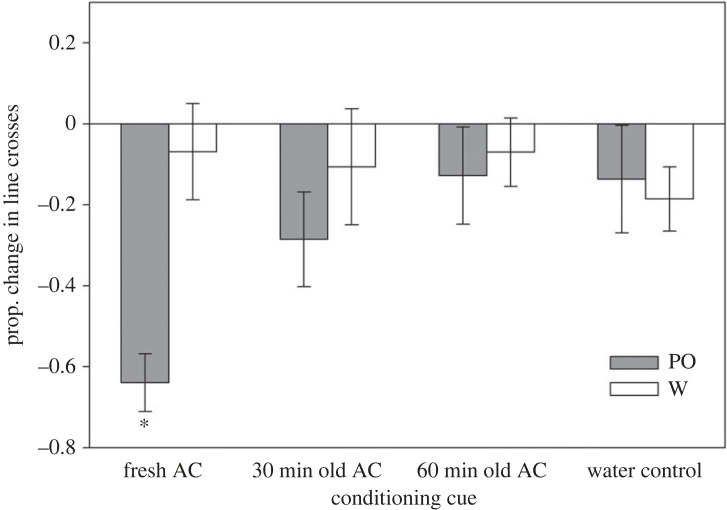
Mean (± s.e.) line crosses by tadpoles in experiment 2 (9-day post-conditioning) when tested with either the PO or blank water (W) after being conditioned with the odour paired with either fresh AC, 30 min old AC or 60 min old AC, or blank water. Asterisks represent significant test-cue comparisons, revealing reduced activity in response to the odour compared to testing with blank water.

When testing for the retention of learned responses at 9-day post-conditioning, we again found a significant interaction between treatment and cue, revealing differential retention across treatments (*F*_1, 44_ = 3.16, *p* = 0.034; figure 2; electronic supplementary material, table S3a). At this point, only tadpoles conditioned with fresh AC continued to show a significant learned response (*F*_1, 14_ = 37.38, *p* < 0.001; electronic supplementary material, table S3b), unlike those conditioned with 30 min old AC (*F*_1, 12_ = 0.35, *p* = 0.57; electronic supplementary material, table S3c), 60 min old AC (*F*_1, 10_ < 0.01, *p* = 0.94; electronic supplementary material, table S3d) and water (*F*_1, 8_ = 0.71, *p* = 0.43; electronic supplementary material, table S3e).

## 4. Discussion

Uncertainty of predation risk poses a major problem for prey that must optimize the balance between predator avoidance and other fitness-enhancing activities. In this study, we manipulated the age of information about predation risk to explore whether prey were uncertain about older information that may no longer be dangerous. We then used the length of the memory window as an objective measure of the prey's perceptual uncertainty. Specifically, tadpole prey first learned to recognize a novel odour as a predator after being exposed to the odour paired with AC that had been aged no longer than 1 h. However, only tadpoles conditioned with fresh AC (less than 5 min old) continued to show learned responses when tested 9-day post-conditioning. While there was less power to detect significant differences on day 9 due to lower sample sizes, the magnitude of the differences between the test cues (PO versus control) in the aged treatments had decreased by over half since day 2, unlike the fresh AC treatment where a significant antipredator response remained (figures 1 and 2). Thus, our statistical results represent a real biological phenomenon of decreased retention of learned responses from aged AC.

Because AC degrade as they age [21,23], the chemical by-products probably become less recognizable over time. This may be analogous to typically weaker responses to heterospecific AC observed among several species, with the response intensity weakening with increasing phylogenetic distance (e.g. [38–40]). Hence, as the chemical signature of the AC changes, so does their recognizability and the uncertainty associated with the information. Exposure to conspecific AC indicates the presence of a predator attack, so fresh AC indicate that the attack was nearby and that the predator is probably still present [12]. A novel odour in the vicinity of fresh AC is probably that of the predator, but a novel odour paired with older (degraded) AC is more likely to be a fortuitous pairing. Given this uncertainty, initially responding to the odour might be appropriate, whereas without reinforcement responding to an uncertain threat may become too costly in the long term [6,41].

We were somewhat surprised that tadpoles showed an initial learned response from 60 min old AC. In a previous study, tadpoles showed a weakening in their response to AC that were aged for 2 h compared to those that were fresh [24]. This may suggest that this weakening begins to occur between 1 and 2 h of ageing, or that the conditions under which the AC degraded in our stimulus buckets were not as impactful as fully natural conditions. AC are known to degrade at different rates depending on the environmental conditions and the source species [21]. Future research identifying the chemical composition of fresh and aged AC, as well as the factors in the natural environment that affect their persistence, is much needed.

The modern world is being shaped by widespread and rapid anthropogenic changes, which often present animals with disrupted or novel information [42]. Indeed, recent projections indicate that range expansions due to climate change will result in hundreds of thousands of first encounters between species [43], including many that will become invasive predators of native prey [44]. Because uncertainty leads to decision errors [5], understanding how prey manage uncertainty about novel predation risk is a pressing issue for behavioural ecologists and conservation scientists alike. Further attention should go toward the causes and consequences of uncertainty in the ecological world, particularly for the role of uncertainty in learning about novel species. While we may never fully know the perceptual world of other species [45], measuring the memory window can give us a glimpse into the important perceptual phenomenon of uncertainty.

## Data Availability

The data are available from the Dryad Digital Repository: (https://doi.org/10.5061/dryad.ngf1vhj03) [46]. Supplementary material is available online [47].
